# Anticancer properties of a defensin like class IId bacteriocin Laterosporulin10

**DOI:** 10.1038/srep46541

**Published:** 2017-04-19

**Authors:** Piyush Baindara, Ankur Gautam, G. P. S. Raghava, Suresh Korpole

**Affiliations:** 1 Microbial Type Culture Collection and Gene bank, CSIR-Institute of Microbial Technology, Chandigarh-160036, India; 2 Bioinformatics Centre, CSIR-Institute of Microbial Technology, Chandigarh-160036, India

## Abstract

Laterosporulin10 (LS10) is a defensin like peptide from *Brevibacillus* sp. strain SKDU10 that inhibited microbial pathogens. However, in this study, anticancer activity of LS10 was examined against different cancer cell lines and compared with normal cells. LS10 displayed cytotoxicity against cancer cells like MCF-7, HEK293T, HT1080, HeLa and H1299 at below 10 μM concentration, but not against prostate epithelium cells RWPE-1. Additionally, no hemolysis was observed at significantly higher concentration compared to IC_50_ values observed for different cancer cell lines. Release of lactate dehydrogenase from cancer cell lines at 15 μM concentration upon 120 min treatment indicated the lytic ability of LS10. Accordingly, electron microscopy experiments also confirmed the necrotic effect of LS10 at 15 μM concentration against cancer cells. Furthermore, flow cytometry analysis of treated cancer cell lines revealed that LS10 induce apoptosis even at 2.5 μM concentration. Nevertheless, RWPE-1 cells remained viable even at 20 μM concentration. These results provide evidence that LS10 is an anticancer bacteriocin, which causes apoptotic and necrotic death of cancer cells at lower and higher concentrations, respectively. Taken all results together, the present study signifies that LS10 is an anticancer peptide that could be further developed for therapeutic applications.

Cancer is one of the leading cause of death worldwide (http://seer.cancer.gov/statfacts) and the frightening statistics underscore the need to examine the novel anticancer agent and modes of therapy^1^,^2^,^3^. The major goal of modern oncology program is to discover better anticancer entities with novel modes of action. Ideally, anticancer drugs should specifically target cancer cells without any toxic effect against normal cells but, unfortunately, most of the available anticancer drugs display severe side effects. Moreover, development of multidrug resistance by cancer cells^4^,^5^ makes the situation even more critical. Therefore, to tackle this grim situation, considerable efforts are being made throughout the world over past several years to discover novel and better therapeutic candidates for cancer therapy. In this context, recent utilization of small peptides in cancer treatment^6^,^7^ has been attracted a lot of scientific attention as cancer therapeutics.

A large number of naturally occurring antimicrobial peptides (AMPs) from various sources have been reported in the literature that displayed anticancer properties^8^,^9^,^10^. In fact, in the recent past, many live or attenuated bacteria were patented as potential anticancer agents^11^,^12^,^13^. Additionally, microbial products including toxins, enzymes, antibiotics, various proteins, peptides and other low molecular weight products have also been evaluated for their anticancer properties^12^,^14^. Various bacterial peptides with antimicrobial activity were also demonstrated activity against cancer cells^15^,^16^,^17^,^18^,^19^, however, only a few of these peptides characterized in detail for anticancer activity^20^,^21^,^22^. AMPs produced by bacteria are relatively amenable to bioengineering and demonstrated considerable therapeutic efficacy^23^,^24^, therefore, such peptides are considered as promising agents for anticancer therapies^19^,^25^,^26^,^27^. Most of these AMPs, also known as bacteriocins, are reported to be non-cytotoxic and non-hemolytic in nature^28^,^29^. Accordingly, AMPs produced by members of the genus *Brevibacillus*, particularly *Brevibacillus laterosporus* produces defensin like antimicrobial peptides^30^, antibiotics like laterosporamine^31^, acyl dipeptides like tupuselei amides, antifungal polyketides like basilisk amides^32^, lipopeptide antibiotic like tauramamide^33^, cyclodecapeptides like laterocidin and its analogues^34^ which inhibits growth of both Gram-positive and Gram-negative bacteria. Additionally, novel thrombin inhibitors like bacithrocins A, B and C^35^ and anticancer antibiotic like spergualin are also reported from strains of *B. laterosporus*^36^.

In a recent study we have characterized a defensin like AMP laterosporulin10 (LS10) that showed antibacterial activity against pathogens like *M. tuberculosis* strain H37Rv and *Staphylococcus aureus*^37^. In fact, it selectively inhibited the growth of *M. tuberculosis* H37Rv at significantly low LD_50_ values (0.5 μM) when compared to *M. smegmatis* MC2 155 in *in vitro* and *ex vivo* assays. Further, insights into the mechanism of action using electron microscopy and flow cytometry experiments assigned it to the membrane permeabilizing bacteriocins. Since, LS10 efficiently killed *M. tuberculosis* H37Rv residing inside the macrophages without any antagonistic activity against macrophages^37^, we further evaluated its anticancer potential and compared with normal cells.

## Results

### LS10 is a defensin like peptide with randomic structure in solution

**Figure 1:**
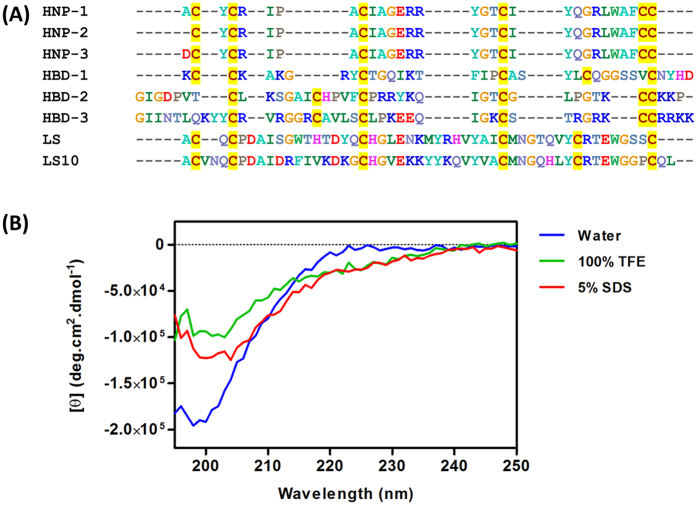
Amino acid sequence alignment of LS10 with LS, human β-defensins, human neutrophil defensins and CD spectra under different conditions. (**A**) Alignment of LS10 with LS, human beta defensins (HBD1, HBD2, HBD3) and human neutrophil defensins (HNP1, HNP2, HNP3), displayed presence of conserved Cysteine residues involved in disulfide bond formation. (**B**) CD spectrum of LS10 under different conditions revealed random coil in solution. CD spectra of LS10 in water (blue line), 100% TFE (green line) and in 5% SDS (red line) is shown. All CD spectra were obtained at pH 7.0 in 10 mM Tris-HCl buffer.

Eukaryotic defensins are multifunctional peptides known to exhibit anticancer properties. Therefore, in order to determine the similarity of defensin like bacteriocin LS10 with known eukaryotic defensins, we selected an earlier reported defensin like bacteriocin laterosporulin (LS), human β-defensins (HBD1, HBD2, HBD3) and human neutrophil defensins (HNP1, HNP2, HNP3) which are active against mammalian cells^38^,^39^,^40^. All the sequences were aligned to compare with LS10 (Fig. 1A). As observed for LS, LS10 also contained six cysteine residues which are involved in disulfide bond formation at conserved positions a characteristic feature of eukaryotic defensins^30^,^37^. Thus, to get more insight into structural aspects, we have performed circular dichroism (CD) for LS10. Interestingly, CD spectrum of the LS10 in water (Fig. 1B) was characterized by the presence of negative values of molar ellipticity (θ) for wavelengths shorter than 200 nm and a minimum close to 200 nm, a characterstic of random coil conformation^41^,^42^. Further, CD spectra obtained in membrane mimicking environments including 5% SDS and 100% TFE were also confirmed the randomic structure of LS10^43^,^44^. However, LS10 might acquire a secondary structure as a negative shift is found to be minimum in 5% SDS or 100% TFE when compared to water. The calculations of percentage or level of helicity (H) obtained from the deconvolution of LS10 concentrations and molar ellipticity (θ) confirm the predominance of randomic structures in 5% SDS and 100% TFE as well (Fig. 1B).

### Cytotoxicity of LS10 towards cancer cells

**Figure 2:**
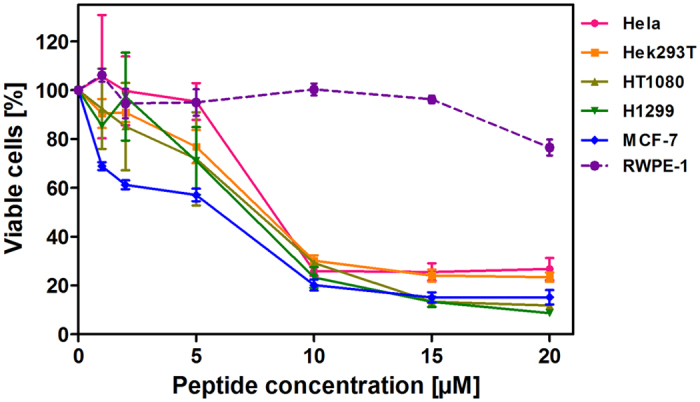
Cell viability assay of human cancer and normal cell lines treated with LS10. HeLa, HEK293T, HT1080, H1299, MCF-7 and RWPE-1 cells were seeded in 96 well plates. Subsequently, cells were incubated for 24 h with increasing doses of LS10 (0–20 μM) and the cell survival was determined by MTT assay. Blank growth medium and 1% Triton X-100 were used as negative and positive controls, respectively. Assay performed in triplicate in three independent experiments. Error bars represent SD. All groups were compared to the controls for statistical significance. P < 0.05 is considered significant.

The anticancer activity of LS10 was examined using five different human cancer cells including HeLa, MCF-7, HT1080, H1299, and HEK293T. The cell lines were incubated with increasing concentration of LS10 (1–20 μM) in their respective growth medium revealed LS10 to be cytotoxic in nature against all cancer cell lines tested (Fig. 2). A dose-dependent cytotoxicity was observed on all cancer cell lines with maximum activity being observed at 10 μM and highest activity against MCF-7 cells. While 40% cytotoxicity was observed on MCF-7 at 5 μM concentration of LS10, only 20% cytotoxicity was displayed by HT1080, HEK293T and H1299. However, no significant cytotoxicity was observed on HeLa cells at 5 μM concentration, but 80% cytotoxicity was observed at 10 μM for HeLa and other cell lines (Fig. 2).

### LS10 showed low toxicity towards normal cells

**Figure 3:**
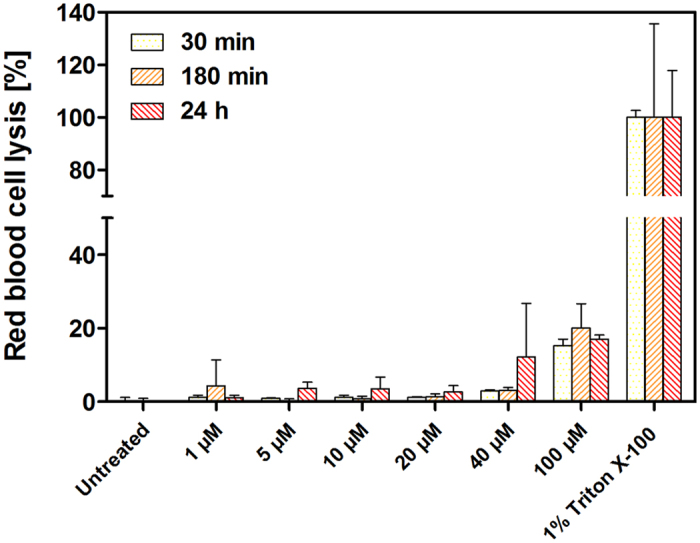
Hemolysis ability of LS10. Hemolysis ability of LS10 was determined using rabbit RBCs. Purified LS10 and RBC samples were prepared in PBS. Assay performed in triplicate in three independent experiments. Error bars represent SD. All groups were compared to the controls for statistical significance. P < 0.05 is considered significant.

To be a good anticancer agent, LS10 essentially should not be toxic towards normal cells. Therefore, we sought to examine the cytotoxic effect of LS10 against normal cells with increasing concentration (1–20 μM) against normal prostate epithelium cells (RWPE-1). The results demonstrated that LS10 did not show any cytotoxicity against normal cells up to 15 μM, while significant cytotoxicity was observed against cancer cell lines at this concentration. At 10 μM concentration, more than 95% of normal cells remained viable while 80% of cancer cells lost viability. However, at 20 μM concentration about 20% of normal cells were found to lose viability (Fig. 2). To examine the cytotoxicity of LS10 against red blood cells (RBCs), if any, we performed hemolysis assay in a time-dependent manner for 30 min, 180 min and 24 h intervals using various concentrations (1–100 μM) of LS10. As shown in Fig. 3, no significant hemolysis was observed up to 40 μM concentration of LS10. Therefore, LS10 was concluded as non-hemolytic bacteriocin as it did not cause hemolysis even at 20 times higher concentration of IC_50_ values against various cancer cell lines (Fig. 3).

### LS10 causes lactate dehydrogenase release from cancer cells

**Figure 4:**
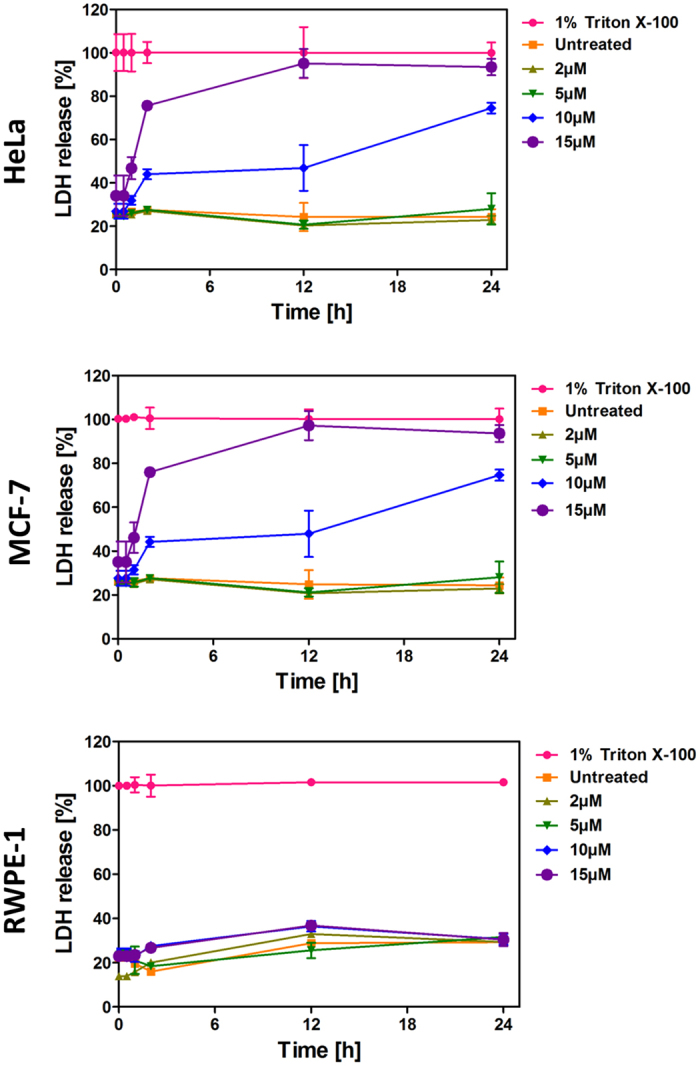
Effect of LS10 on LDH release in HeLa, MCF-7 and RWPE-1 cells. HeLa, MCF-7 and RWPE-1 cells were incubated in a time dependent manner (0–24 h) with increasing concentrations (0–15 μM) of LS10. Blank growth medium and 1% Triton X-100 was used as negative and positive control, respectively. Assay performed in triplicate in three independent experiments. Error bars represent SD. All groups were compared to the controls for statistical significance. P < 0.05 is considered significant.

To evaluate the activity of LS10 on membrane integrity of cancer and normal cells, we have carried out lactate dehydrogenase (LDH) release assay with HeLa, MCF-7 and RWPE-1 cells. For this, cells were incubated with increasing concentration of LS10 (2, 5, 10 and 15 μM) and LDH release was monitored up to 24 h at regular time intervals. As shown in Fig. 4, significant LDH release was not observed in HeLa and MCF-7 cells up to 60 min of incubation with all tested concentrations of LS10. However, 50% and 75% increase in LDH release was observed for HeLa and MCF-7 cells respectively, after 120 min of incubation with 15 μM concentration of LS10 (Fig. 4). These results are in accordance with the cell viability assay that displayed significant cytotoxicity against HeLa and MCF-7 cells at 15 μM concentrations of LS10. Interestingly, no LDH release was observed in RWPE-1 cells even after 24 h of incubation at 15 μM concentration of LS10 (Fig. 4).

### LS10 acts on cell membrane of cancer cells

**Figure 5:**
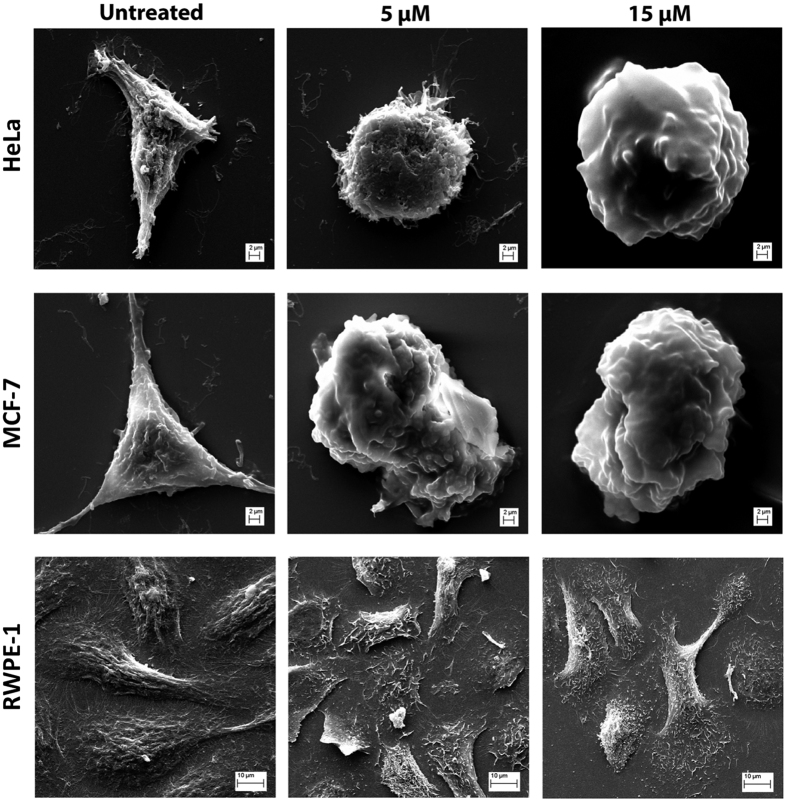
Scanning electron microscopy of HeLa, MCF-7 and RWPE-1 cells treated with LS10. Scanning electron microscopy micrographs of HeLa, MCF-7 and RWPE-1 cells treated with LS10. Cells in PBS (untreated) and cells after treatment with 5 and 15 μM concentrations of LS10 are shown under respective panel. Untreated cells in PBS were used as control. Suspension of cells and purified LS10 samples were prepared in PBS.

To further confirm the results of LDH release and MTT assays, scanning electron microscopy was performed using HeLa and MCF-7 cancer cells along with RWPE-1 normal cells treated with LS10. We have primarily observed changes in cell morphology, microscopically upon treatment with LS10 (Figure S1), which were further confirmed by electron microscopy performed under the identical conditions against cancer and normal cells. As shown in Fig. 5, MCF-7 cells treated with 5 μM concentration of LS10, showed significant alterations on the cell membrane. However, HeLa cells displayed profound effect at 15 μM but not at 5 μM concentration of LS10. As a result of LS10 effect, cell membrane of MCF-7 cells became smooth and microvilli were lost from the cell surface even at 5 μM concentration but in case of HeLa cells, the microvilli were completely vanished at 15 μM concentration of LS10 (Fig. 5). On the other hand, no such effect was observed on RWPE-1 cells even after 3 h of treatment with LS10 (Fig. 5). These results were in agreement with the MTT and LDH release assay.

### LS10 induces apoptosis in cancer cells

**Figure 6:**
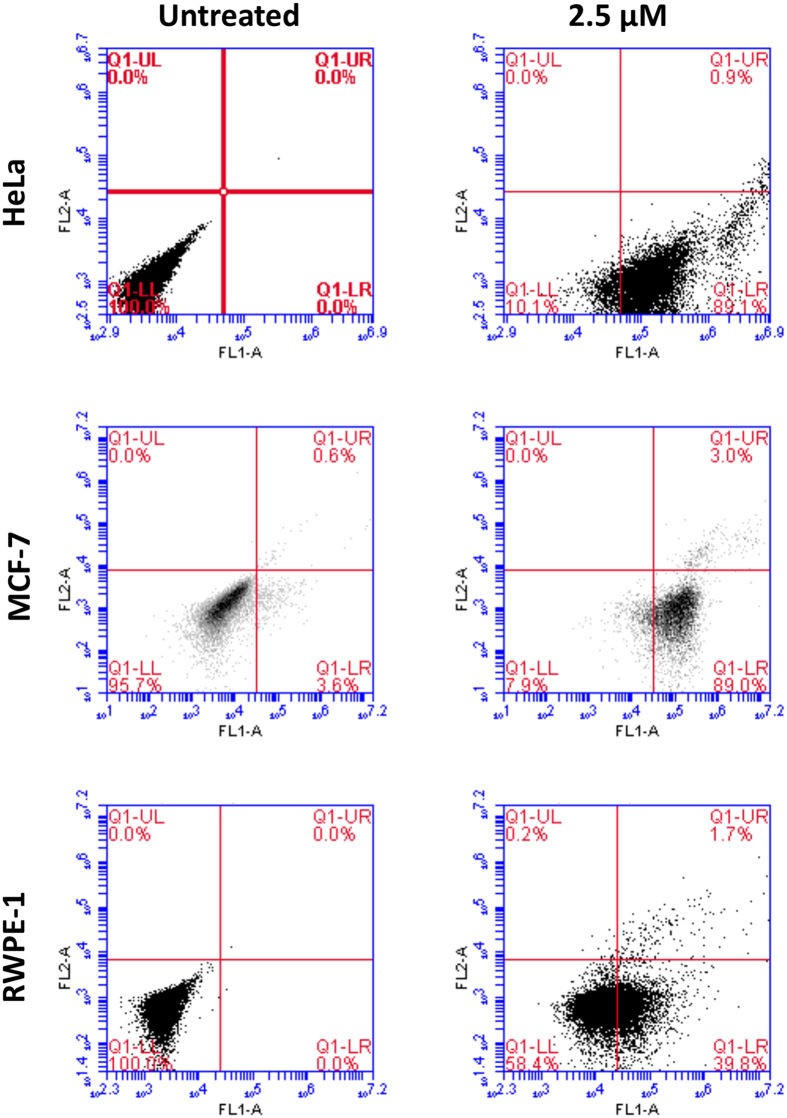
LS10 induced apoptosis in HeLa, MCF-7 and RWPE-1 cells. HeLa, MCF-7 and RWPE-1 cells were incubated with 2.5 μM concentrations of LS10 for 2 h and subsequently trypsinized and washed with ice cold PBS. Cells were stained with Annexin V/PI and analyzed on flow cytometer. Cells in PBS without LS10 treatment and cells after treatment with 2.5 μM concentration of LS10 are shown under respective panel. Experiment performed in triplicate in three independent experiments.

In order to examine the possibility of LS10 to exhibit any other mechanisms of action, we have evaluated its ability to induce apoptosis in cancer and normal cells. Thus, HeLa, MCF-7 and RWPE-1 cells were incubated with sub-lethal concentration (2.5 μM) of LS10 for 2 h and 24 h. The induction of apoptosis was examined by Annexin V/PI staining followed by flow cytometry analysis. Results clearly demonstrated that LS10 induced apoptosis in cancer cells as approximately 90% of HeLa and MCF-7 cells were found to be Annexin V positive after 2 h of treatment when compared to untreated cells (Fig. 6). However, only about 40% RWPE-1 cells were found to be Annexin V positive (Fig. 6). Results after 24 h of treatment with LS10 (Figure S2) were also followed the same pattern as observed for 2 h treatment. These results suggest that LS10 induces apoptosis in cancer cells even at very low concentration and less toxic towards normal cells such as RWPE-1.

## Discussion

LS10, a defensin like class IId bacteriocin, was isolated from *Brevibacillus* sp. strain SKDU10 and found to inhibit microbial pathogens but did not found cytotoxic towards macrophages^37^. Therefore, in the present study we have made an attempt to explore the anticancer potential of defensin like bacteriocin LS10. Sequence alignment of LS10 with other known anticancer defensins demonstrated that cysteine residues are conserved in position with LS that showed typical disulfide bonding pattern with human β-defensins, a key feature defensin like peptides.

So, in order to examine the anticancer activities of LS10, cell viability assays were performed with different cancer cell lines along with normal cell line. LS10 displayed significant cytotoxicity against cancer cell lines with comparable MIC values found against bacterial cells^37^. Though studies revealed LS10 to be effective against all tested cancer cell lines with maximum potency against MCF-7 and low cytotoxicity against normal cells, further to get more insights, hemolytic properties of LS10 was also tested at different time points (30 min, 180 min and 24 h). Interestingly, LS10 did not show any significant differences in hemolysis at prolonged durations when compared to values obtained during the earlier study^37^. It is pertinent to mention that most of the AMPs, despite their high anticancer activities, could not move forward in drug development pipeline because of their high hemolytic nature^8^,^45^.

The surface of cancer cells is different from the normal cells in many ways^46^ and one of the major difference is found to be in surface charge. Cancer cells are relatively more negatively charged compared to the normal cells due to the high expression of negatively charged lipid molecules^47^,^48^. Therefore, most of the cationic AMPs display broad spectrum of anticancer activities while their selective activity against cancer cells is due to the net high positive charge^49^,^50^. AMPs show diverse mechanisms of action against cancer cells, while most of them act on cell membrane, a few others cause the disintegration of mitochondrial membranes also^19^,^49^,^51^. Nisin and its variants are commonly used as a food preservative, and recently they have been reported to induce apoptosis, cell cycle arrest, and inhibition of cell proliferation in HNSCC (Head and neck squamous cell carcinoma) cells^19^,^25^. The mitochondrial membrane is believed to originate from endosymbiotic prokaryotes and having high similarities with bacterial cell membranes, which is justified by mitochondrial membrane specific activity of AMPs^19^,^51^,^52^. In fact, eukaryotic defensins are also reported to have antimicrobial and anticancer activities^9^,^53^,^54^. Thus, in order to determine defensin like bacteriocin LS10 mediated cancer cell cytotoxicity by membrane disintegration, we have performed LDH release assay, but at 2 to 5 μM concentrations it did not trigger any LDH release. However, at 10 and 15 μM concentrations, LS10 caused significant LDH release after 120 min of treatment in HeLa and MCF-7 cells, suggesting the loss of membrane integrity. These observations were also confirmed by electron microscopy. Interestingly, RWPE-1 did not show any LDH release or adverse effect at 15 μM concentration of LS10 even after 24 h of treatment. Taken all these results together, it can be concluded that LS10 mediated cancer cell cytotoxicity is mainly due to membrane disintegration.

Despite the membrane disintegration many AMPs have been reported to induce apoptosis in cancer cells^52^,^55^,^56^. Similarly, LS10 also induce apoptosis in cancer cells. In fact, LS10 was found to be highly apoptotic for cancer cells at low concentration (2.5 μM) and a multi-action bacteriocin, which induces both apoptosis and necrotic effects when treated with lower and higher concentrations, respectively. It is interesting to note that at 2.5 μM concentration, despite the induction of high apoptosis (90%) in HeLa cells no significant cell death was observed in MTT assay. As synthetic anticancer drugs are very expensive and considering the production of bacteriocin LS10 produced by bacteria with novel antibacterial and anticancer activities against human cancer cells, indicates its potential for applications when compared to eukaryotic/human defensins. In fact, production of small AMPs naturally from bacteria is highly desirable and beneficial to test against cancer cells^23^,^57^. In summary, the bacteriocin LS10 is a potential novel anticancer molecule that displayed no adverse effect against normal cells like RWPE-1 and did not cause hemolysis.

## Material and Methods

### Cell cultures

Human cervical cancer cell line, HeLa and human normal prostate epithelium cell line RWPE-1 were obtained from American Type Culture Collection (ATCC, Manassas, VA). Other cell lines like human embryonic kidney cancer cell line (HEK293T), human fibro-sarcoma cell line (HT1080), human lung carcinoma (H1299) and human breast cancer cell line (MCF-7) were gifted by Prof. R.N.K. Bamezai, National Centre of Applied Human Genetics, New Delhi, India. HeLa, HEK293T, HT1080 and MCF-7 cells were grown in DMEM medium (Invitrogen, USA) supplemented with 10% fetal bovine serum (Invitrogen, USA) and 1% penicillin-streptomycin cocktail (Sigma, USA). RWPE-1 cells were grown in keratinocyte serum-free medium supplemented with recombinant human epidermal growth factor and bovine pituitary extract (Invitrogen, USA). All cells were maintained in CO_2_ incubator (ThermoScientific, USA) at 37 °C and 5% CO_2_ environment.

### LS10 peptide

The peptide was extracted from *Brevibacillus* sp. strain SKDU10 and purified as mentioned previously^37^. Amino acid sequence of LS10 was compared and aligned with laterosporulin and other human defensins with anticancer properties using Bioedit software (http://www.mbio.ncsu.edu/bioedit/bioedit.html).

### Circular dichroism

CD experiments were performed using a Jasco 815 spectropolarimeter (JASCO, USA), coupled to a Peltier Jasco PTC-423L system for temperature control. HPLC purified LS10 (0.05 mg/ml) in water was used for the experiments carried out in the presence of SDS micelles (5% SDS) (BioRad, USA) and 2,2,2-trifluoroethanol (100% TFE) (Sigma, USA). LS10 samples were also prepared to a final concentration of 0.05 mg/ml in 10 mM Tris-HCl (pH 7.0) for both SDS and TFE experiments. Spectra were collected and averaged over six scans in the spectral range of 195–250 nm, with 0.2 cm path length quartz cells (Starna Scientific, USA) at 37 °C. Parameters used in experiments were 0.2 nm step resolution, 50 nm/min speed, 1 s response time and 1 nm bandwidth. Following baseline correction, the observed ellipticity, θ (m degree) was converted to the molar ellipticity [θ] (degree.cm^2^.dmol^−1^)^43^.

### Cell viability assay

Cell viability was determined by using MTT assay. Briefly, HeLa, HEK293T, HT1080, H1299, MCF-7 and RWPE-1 cells (5 × 10^3^ cells/well) were seeded in 96 well plates. Upon 24 h of incubation, the growth medium was replaced with fresh medium containing increasing concentrations of LS10 (1–20 μM). While 1% Triton X-100 (G Biosciences, USA) was used as positive control, blank growth medium as negative control. The plates were incubated for additional 24 h and each well was added with 20 μl of MTT solution (5 mg/ml in PBS) and incubated for 3 h at 37 °C. Subsequently, the MTT containing medium was removed and 50 μl of Dimethyl sulfoxide (Sigma, USA) was added to each well. To assess the percentage of live cells in samples, absorbance was read at 590 nm on ELISA plate reader (ThermoScientific, USA).

### LDH release assay

The LDH release assay was performed using CytoTox 96 Non-Radioactive Cytotoxicity Assay kit (Promega, USA). Briefly, HeLa, MCF-7 and RWPE-1 cells (about 5 × 10^3^ cells/well) were seeded in 96 well plates (BD Falcon, USA). Upon 24 h incubation, culture medium was replaced with fresh medium containing LS10 (2, 5, 10 and 15 μM). Blank growth medium was used as negative control and 1% Triton X-100 as positive control. After, 30 min, 60 min, 120 min and 24 h of treatment with different concentrations of LS10, culture medium was collected and centrifuged. Aliquots of 50 μl from each reaction were incubated with 50 μl of reaction buffer supplied with CytoTox 96 Non-Radioactive Cytotoxicity Assay kit (Promega, USA). After 30 min incubation in dark at room temperature, 50 μl stop solution was added to each reaction well and the release of LDH was measured by reading absorbance at 490 nm using ELISA plate reader (ThermoScientific, USA). Cytotoxicity of LS10 was determined by comparing LDH release with the positive control (1% Triton X-100 treated cells).

### Electron microscopy

HeLa, MCF-7 and RWPE-1 cells (about 10^6^ cells/well) were seeded on poly-lysine (Sigma, USA) coated hemocytometer cover slips, placed in 24 well plates (BD Falcon) and incubated in 5% CO_2_ environment at 37 °C temperature. After 24 h, growth medium was replaced with medium containing LS10 (5 and 15 μM, in triplicate). Cells without peptide treatment served as negative control. After 3 h treatment with LS10, cells were washed thrice with ice cold PBS to remove all unattached cells and fixed in modified karnovsky’s fixative for 2 h. Subsequently, cells were treated with OsO_4_ (Sigma, USA) for 1 h. Further, cells were dehydrated in graded ethanol sequentially with 30 min incubation for each step at 4 °C (30–100%). Ethanol dehydrated samples were freeze-dried and processed further as mentioned earlier^58^. Cover slips were placed on aluminium stubs using silver paint and sputter coated with gold. Cells were then observed and photographed using S-260, Leica Cambridge, scanning electron microscope (BRUKER, USA).

### Apoptosis assay

Apoptosis assay was performed using apoptosis assay kit (Invitrogen, USA). Briefly, HeLa, MCF-7 and RWPE-1 cells were seeded in 24 well tissue culture plates (about 2 × 10^5^ cells/well) and incubated for 24 h. Subsequently, culture medium was replaced with fresh medium containing 2.5 μM of LS10 along with a negative control (cells without peptide). Apoptosis induced by the peptide was examined by Annexin V/PI staining followed by flow cytometry analysis of stained cells. Briefly, after 2 h and 24 h of incubation with LS10, cells were harvested, washed twice with cold PBS and resuspended in 1X Annexin binding buffer. Cells were then transferred to fresh 1.5 ml tube containing 100 μl of binding buffer and 5 μl of FITC-conjugated Annexin V and 1 μl of PI were added. The cells were vortexed gently and incubated for 15 min at room temperature in dark. After incubation, 200 μl of 1X Annexin binding buffer was added to each tube and stained cells were analyzed by Accuri flow cytometry (BD Biosciences, USA) with CellQuest software (BD Biosciences, USA) used for analysis of the results.

### Statistical analysis

All comparisons were based on the mean +/− standard deviation of the mean (SD). Parametric data were analyzed using two-way analysis of variance (ANOVA) with the Bonferroni posttest method for comparison between groups. Column statistics for nonparametric data were analyzed by using the D’Agostino and Pearson omnibus normality test along with a one-sample *t* test. Results were considered significant when P values were <0.05. All experiments were performed independently three times in triplicate.

## Additional Information

**How to cite this article:** Baindara, P. *et al*. Anticancer properties of a defensin like class IId bacteriocin Laterosporulin10. *Sci. Rep.* **7**, 46541; doi: 10.1038/srep46541 (2017).

**Publisher's note:** Springer Nature remains neutral with regard to jurisdictional claims in published maps and institutional affiliations.

## Supplementary Material

Supplementary Information
