# Supplementary material for: Anticancer properties of a defensin like class IId bacteriocin Laterosporulin10
Source: Sci Rep. 2017 Apr 19;7:46541. doi: 10.1038/srep46541 (PMC5396196; doi:10.1038/srep46541)

Anticancer properties of a defensin like class IId bacteriocin Laterosporulin10

Piyush Baindara[1], Ankur Gautam[2], GPS Raghava[2] and Suresh Korpole[1†]

Supplementary information

Figure S1: Determination of cytotoxic effect of LS10 on HeLa and MCF-7 cells by using bright field microscopy. Untreated cells in PBS were used as control. Mammalian cells samples and purified LS10 samples were prepared in PBS.

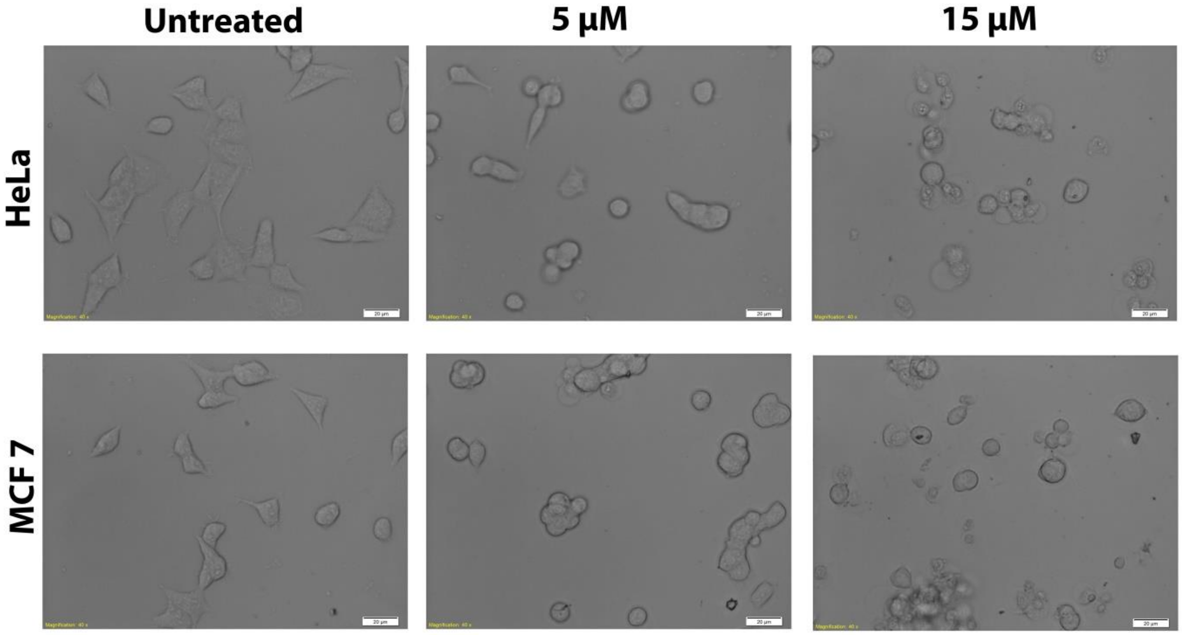

Figure S2. LS10 induced apoptosis in HeLa, MCF-7 and RWPE-1 cells. HeLa, MCF-7 and RWPE-1 cells were incubated with 2.5 µM concentrations of LS10 for 24 h and subsequently trypsinized and washed with ice cold PBS. Cells were stained with Annexin V/PI and analyzed on flow cytometer. Cells in PBS without LS10 treatment and cells after treatment with 2.5 µM concentration of LS10 are shown under respective panel.

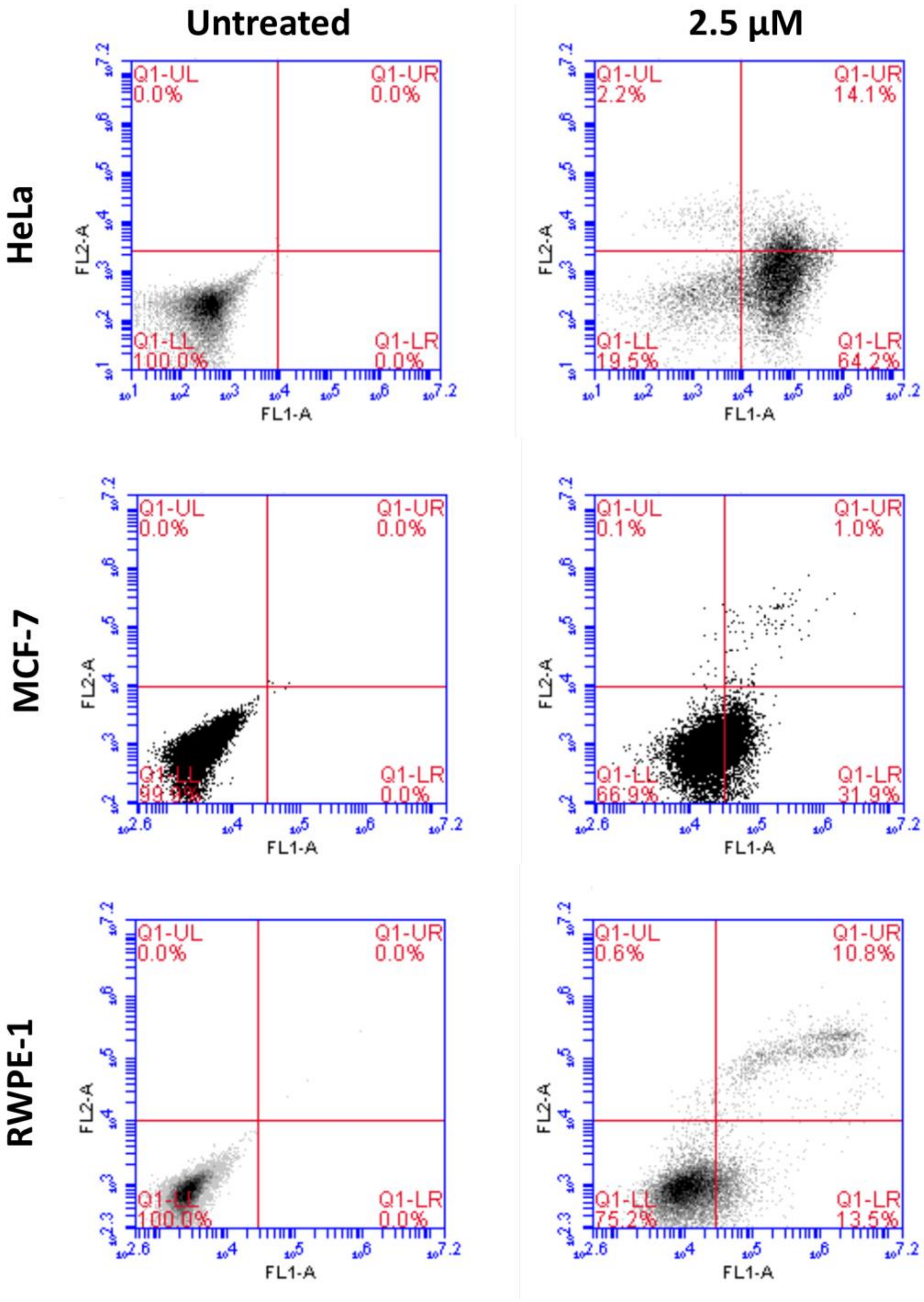

Supplement: Supplementary Information [file srep46541-s1.pdf]
